# Low-dose methotrexate adverse reaction risk in renal impairment: pharmacovigilance and physiological pharmacokinetic model assessment

**DOI:** 10.3389/fphar.2025.1703557

**Published:** 2025-11-06

**Authors:** Lichang Zhang, Jianling Li, Xin Wang, Yaolei Zhang, Sen Gao, Deshi Dong, Yanna Zhu, Shilei Yang

**Affiliations:** 1 Department of Pharmacy, First Affiliated Hospital of Dalian Medical University, Dalian, China; 2 College of Pharmacy, Dalian Medical University, Dalian, China

**Keywords:** low-dose methotrexate, pharmacovigilance, physiologically based pharmacokinetic model, renal impairment, chronic kidney disease

## Abstract

**Objective:**

Low-dose methotrexate (LD-MTX), a treatment regimen involving weekly doses ≤20 mg, is widely used in rheumatoid arthritis. Methotrexate (MTX) is primarily excreted via the kidneys. However, the assessment protocol for the adverse reaction risk threshold of the LD-MTX dosing regimen in renal impairment remains inadequate. This study aims to use pharmacovigilance analysis and physiologically-based pharmacokinetic (PBPK) model to combine the analysis of the risk of adverse reactions of LD-MTX in patients with renal impairment.

**Methods:**

Collected and analyzed disproportionate signals from adverse reaction reports on MTX in patients with renal impairment from the FDA Adverse Event Reporting System (FAERS) from Q1 2004 to Q3 2024. The restricted cubic spline (RCS) model explored the nonlinear relationship between MTX maximum plasma concentration and dose to derive risk thresholds. The PBPK model was developed and validated using MTX data in healthy adults, and further extended to chronic kidney disease (CKD) populations to simulate dose risks.

**Results:**

FAERS analysis revealed heightened risks of hematological disorders, hepatic impairment, and pulmonary adverse events (AEs) with MTX in renal impairment. The optimized threshold based on RCS and the PBPK model simulation results indicated that the risk of adverse reactions increased starting from CKD stage 2.

**Conclusion:**

LD-MTX confers increased adverse reaction risks in renal impairment, notably from CKD stage 2 or higher, necessitating dose adjustments and vigilant monitoring.

## 1 Introduction

Low-dose methotrexate (LD-MTX) is typically defined as a therapeutic dose of ≤20 mg/week (Solomon et al., 2020). Methotrexate (MTX) is one of the most commonly used medications for treating rheumatoid arthritis and has been demonstrated to be the most effective and fastest-acting antirheumatic drug (Torres et al., 2022). However, concerns regarding its safety have persisted since its introduction in 1962 (Weinblatt, 2013). The most frequent adverse reactions include: gastrointestinal reactions, liver dysfunction, skin disorders, blood-related disorders, and nephrotoxicity. In some cases, pulmonary toxicity and bone marrow suppression may also occur (Wang et al., 2018; Weinblatt, 1985). A statistical study found that 72.9% of patients experienced at least one adverse reaction when using LD-MTX to treat rheumatoid arthritis (Torres et al., 2022). MTX is primarily excreted via the kidneys after oral absorption. Over 80% of MTX is excreted unchanged in urine, while a small portion of MTX and its metabolites is excreted through the liver and bile (Maksimovic et al., 2020). As a drug primarily excreted by the kidneys, changes in renal function may amplify the risk of these adverse reactions. Multiple studies have demonstrated a significantly increased risk of adverse reactions when MTX is used in patients with renal impairment. In a study involving 5,648 rheumatoid arthritis patients receiving oral MTX, the incidence of hematologic toxicity among patients with chronic kidney disease (CKD) was 37.5% (33/88), whereas the incidence among non-CKD patients was only one-third that of CKD patients (10.7% [594/5,560]) (Mitsuboshi, 2021). Furthermore, according to reports by Muanda et al., the use of LD-MTX in elderly patients with chronic kidney disease carries a higher risk of toxicity (Muanda et al., 2023). Meanwhile, approximately 13.4% of the global population suffers from CKD. Since early-stage CKD is clinically asymptomatic, the actual number of CKD patients may be significantly higher than current statistics indicate (Hill et al., 2016; Obrador and Levin, 2019). Although existing studies have confirmed that LD-MTX increases the risk of adverse reactions in patients with CKD, the assessment protocols for determining the adverse reaction risk thresholds of LD-MTX dosing regimens across different stages of CKD remain inadequate. Therefore, refining the assessment protocols for the risk of adverse reactions associated with LD-MTX use in patients with renal impairment, particularly through evaluating and determining risk thresholds based on dosage and degree of renal impairment, is an urgent issue requiring resolution.

**FIGURE 1:**
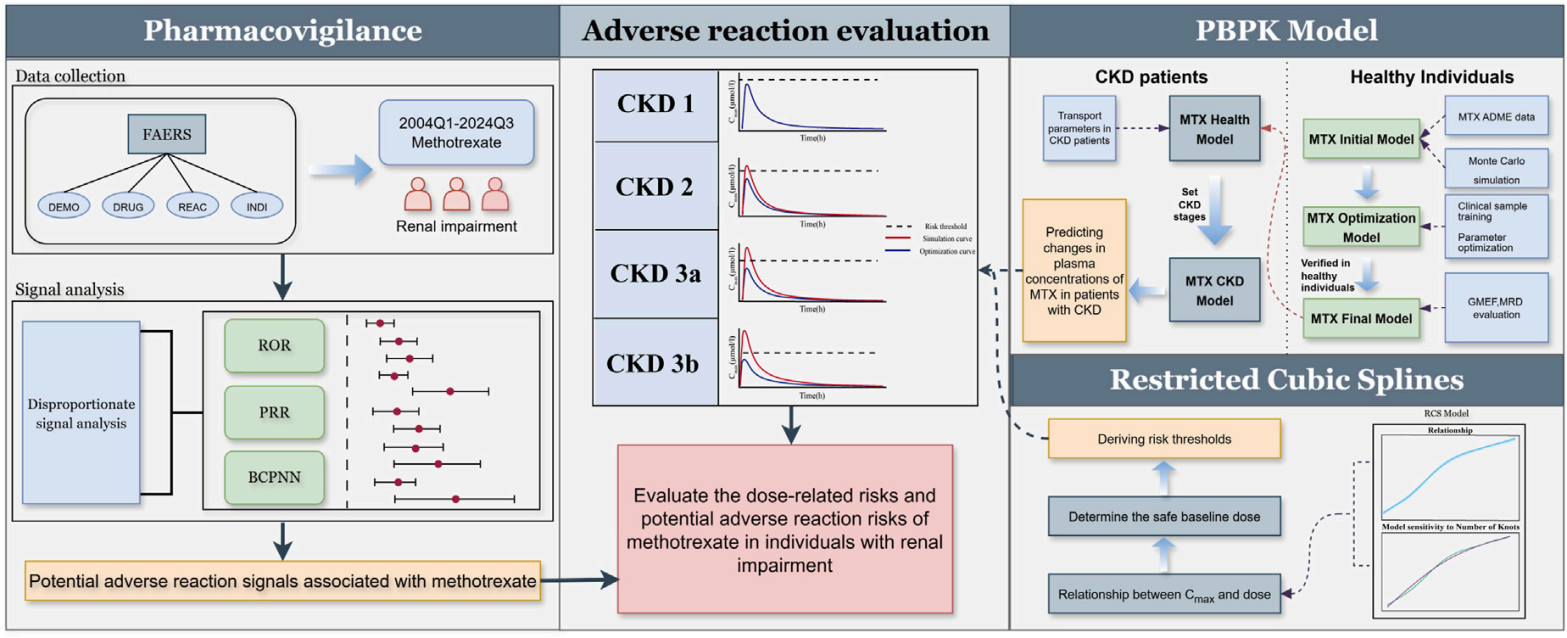
Adverse reaction assessment process for LD-MTX in patients with renal impairment. Through pharmacovigilance data analysis, adverse reaction signals associated with MTX use in patients with renal impairment were identified. Based on existing clinical study results, an MTX PBPK model was established and validated in healthy populations and extended to CKD populations. The relationship between MTX maximum plasma concentration and dose was explored using restricted cubic spline model, and risk thresholds for CKD populations were derived to assess the risks associated with different doses across various stages of CKD.

In recent years, research utilizing data mining and pharmacovigilance analysis from the U.S. Food and Drug Administration Adverse Event Reporting System (FAERS) has steadily increased. Its advantages lie in the vast volume of adverse reaction reports and the real-world data on the clinical safety of prescription drugs (Bone and Houck, 2017; Morris et al., 2024). The FAERS database provides MTX-related case data that effectively assesses the correlation between adverse reactions and LD-MTX use in patients with renal impairment. Understanding the pharmacokinetic changes of LD-MTX in patients with renal impairment is crucial for evaluating its safety. Studies indicate that MTX pharmacokinetic alterations are closely associated with adverse reaction risks, particularly in patients with impaired renal function (Grim et al., 2003). Therefore, employing physiologically based pharmacokinetic (PBPK) models to investigate the absorption, distribution, metabolism, and excretion of MTX in humans is an appropriate approach. Concurrently, recent years have seen growing advocacy for utilizing PBPK models to explore the impact of renal impairment on pharmacokinetics (ResearchCenter for Drug Evaluation and, 2024). In this study, we first analyzed MTX cases within the pharmacovigilance database to identify potential adverse reaction signals in patients with renal impairment. Subsequently, we established a PBPK model for MTX to further explore the dose-risk relationship across varying degrees of renal impairment. The combined assessment of pharmacovigilance and PBPK modeling can better elucidate the safety of LD-MTX use in patients with renal impairment and provide a more comprehensive risk assessment approach for clinical practice, as illustrated in Figure 1.

## 2 Materials and methods

### 2.1 Data sources

The FAERS database is a public, free pharmacovigilance database that is updated quarterly with adverse event data. The official portal of the FDA provides data in two formats: ASCII and XML. To improve computational efficiency, we obtained ASCII data files spanning from Q1 2004 through Q3 2024. Pharmacovigilance data can be found here: https://fis.fda.gov/extensions/FPDQDE-FAERS/FPD-QDE-FAERS.html.

The plasma concentration-time curve for MTX was retrieved from a series of published clinical studies involving healthy volunteers and digitized using OriginPro 2024 (OriginLab Corporation, Northampton, MA, United States). Part of the clinical study dataset from healthy volunteers was used to build the PBPK model, while the remainder supported model evaluation and testing. Detailed information is provided in the Electronic Supplementary Material.

### 2.2 Pharmacovigilance data extraction and analysis

In this study, four datasets (DEMO, DRUG, INDI, REAC) were cleaned and merged. First, AEs in the FAERS database are coded according to the Medical Dictionary for Regulatory Activities (MedDRA) terminology (https://www.meddra.org/). In the INDI file, we used the MedDRA high-level terms “Renal failure and impairment” (Version 26.1, code: 10038443) and “Complications of renal failure” (Version 26.1, code: 10010180) to screen all adverse reaction indications under this term hierarchy. After screening and deduplication, the INDI data is mapped to the REAC file using the PRIMARY ID and CASE ID fields to obtain complete adverse reaction and indication information. For the DRUG dataset, FAERS, as a spontaneous reporting system, may record drug names using multiple names, such as generic names, chemical structure names, brand names, abbreviations, or even incorrect names (Khaleel et al., 2022). Therefore, to standardize drug names, we used the Medical Concept Extraction System for Unstructured Information Management Architecture (MedEx-UIMA) to uniformly map drug names to generic drug names (Jiang et al., 2014). This system enables the extraction and standardized coding of drug names and can map them using RxNorm drug codes to ensure the standardization of drug names. Subsequently, the standardized DRUG dataset was mapped to the cleaned INDI and REAC datasets using PRIMARY ID and CASE ID, followed by deduplication. Finally, the merged dataset from the previous step is mapped against the DEMO dataset using PRIMARY ID and CASE ID to deduplicate, to get the complete adverse reaction cases, rows with missing AE information will be treated as null values and excluded from statistical analysis. According to MedDRA (Version 26.1), all AEs are coded as Preferred term (PT) and classified by System Organ Class (SOC). And PTs unrelated to drug adverse reactions are excluded, including product issues, social environment, various injuries, poisoning, surgical complications, and medical procedures. All the above work was completed using Python 3.11 (Python Software Foundation, Wilmington, United States) and Microsoft Excel (Microsoft Company, Microsoft Build 16.0.17932.20408).

For signal detection, we extracted MTX-related reports and applied the Proportional Reporting Ratio (PRR), Reporting Odds Ratio (ROR), and Bayesian Confidence Propagation Neural Network (BCPNN) to identify adverse reaction signals. PRR estimates relative risk, with a signal detected if frequency ≥3, PRR ≥2, and chi-squared ≥4; however, it is sensitive to false positives with low case numbers. ROR, a less biased estimate of risk ratio, detects a signal if frequency ≥3 and the 95% CI lower limit >1. BCPNN remains stable with small case numbers, detecting a signal if frequency ≥3 and the 95% CI lower limit (IC_025_) >0 (Noguchi et al., 2021; Trillenberg et al., 2023). Therefore, we combined PRR, ROR, and BCPNN for signal detection. Higher PRR, ROR, or IC values indicate stronger signals. A PT was considered a signal if it met all three methods’ criteria.

### 2.3 Development of PBPK models for methotrexate in healthy individuals

**FIGURE 2:**
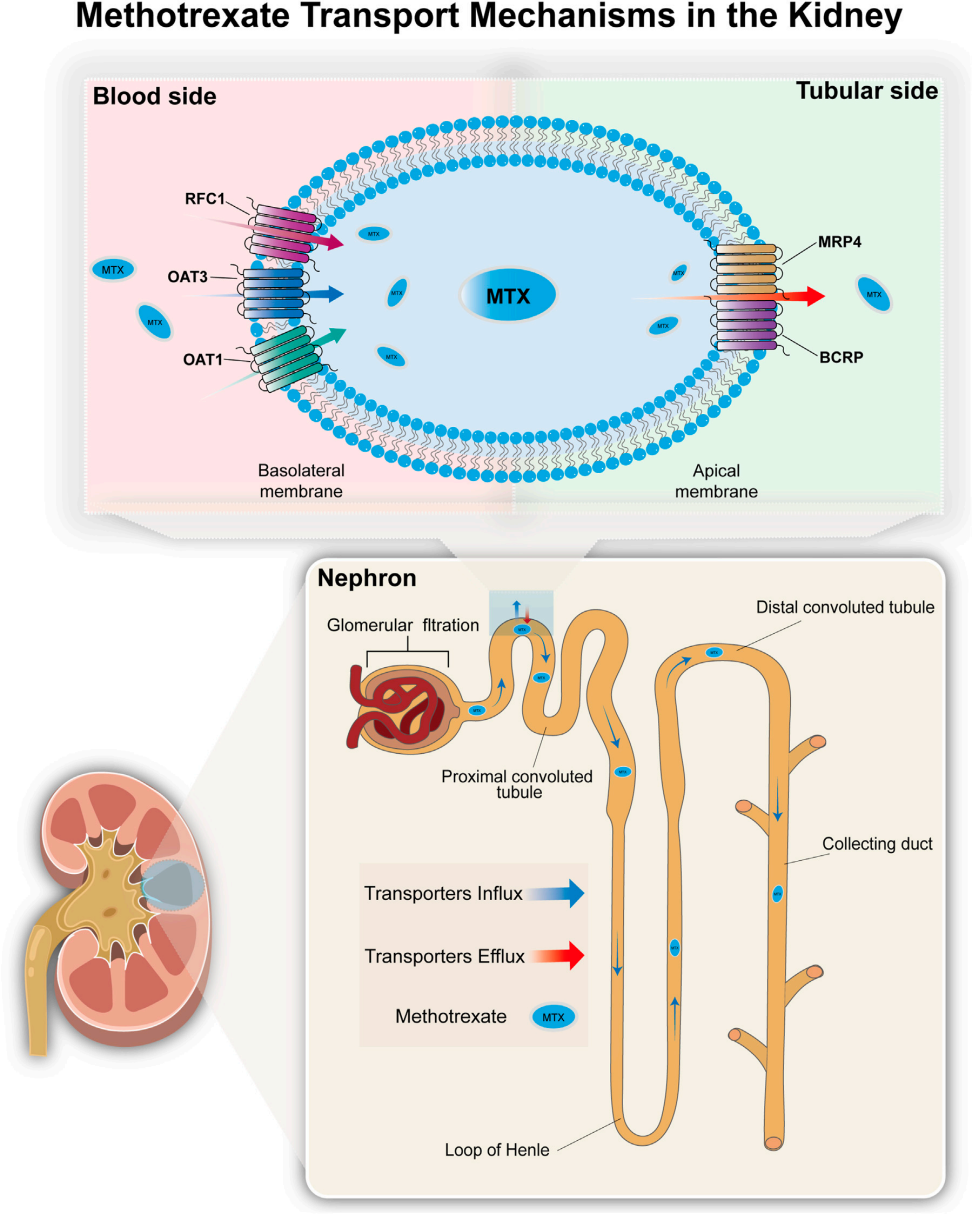
The transport mechanism of methotrexate in the kidneys. Most MTX in the blood enters the renal tubule lumen via glomerular filtration. MTX is actively transported into the proximal tubule epithelial cells via organic anion transporters (OAT1 and OAT3) and reduced folate carri-er (RFC1) located on the basolateral membrane, and then secreted into the renal tubule lumen via efflux transporters (MRP4 and BCRP) located on the apical membrane of the tubule epithelial cells.

The methotrexate PBPK model was developed using PK-Sim^®^ (Open Systems Pharmacology Suite 11.3, www.opensystems-pharmacology.org). MTX is metabolized in the liver, but the vast majority of MTX is excreted via the kidneys. Therefore, for establishing the initial model, we primarily considered MTX’s glomerular filtration and tubular secretion in the kidneys. Regarding tubular secretion, it mainly involves basolateral membrane uptake and apical membrane efflux (Grim et al., 2003). Organic anion transporter 1 (OAT1), organic anion transporter 3 (OAT3), and reduced folate carrier 1 (RFC1) in the basolateral membrane of proximal tubule cells transport MTX from blood to renal tubule epithelial cells (Uwai et al., 2004; Takeda et al., 2002; Wang et al., 2001; Wright et al., 2022). Multidrug resistance-associated protein 4 (MRP4) and breast cancer resistance protein (BCRP) mediate the apical membrane efflux of MTX, secreting it from epithelial cells into the renal tubule lumen for excretion (Chen et al., 2025; Huls et al., 2008; Ivanyuk et al., 2017). The detailed mechanism is shown in Figure 2. After determining the preliminary transport mechanism, we optimized multiple parameters simultaneously using PK-Sim®’s parameter identification module to refine uncertain values. And we used the Monte Carlo algorithm to optimize parameters, minimizing the difference between simulated and observed pharmacokinetic data. Sensitivity analysis revealed the contribution of each transport carrier to renal excretion. Based on the preliminary results of the transporter contribution sensitivity analysis, as well as the fact that OAT3 (IC_50_ = 61.5 μM) has a significantly higher affinity for MTX than OAT1 (IC_50_ = 998 μM) (Uwai and Iwamoto, 2010). The final hypothesis is that OAT3 and RFC1 mediate absorption in the basolateral membrane of proximal renal tubule cells, while MRP4 and BCRP mediate excretion in the apical membrane of renal tubules, thereby explaining MTX secretion in the renal tubules. At the same time, considering the loss of renal transporters and non-renal clearance effects caused by mapping the model to the CKD population, we introduced partial hepatic clearance to simulate non-renal clearance effects and bile excretion to simulate the metabolic compensation effects of patients with mid-stage CKD (Grim et al., 2003). Finally, MTX’s absorption, distribution, metabolism, and excretion (ADME) data were compiled to optimize model parameters. The PK-Sim^®^ physiological database provided anatomical and physiological data (Kuepfer et al., 2016), with parameters such as age, weight, and height adjusted based on collected demographic data, while others retained default values.

### 2.4 Development of PBPK model for methotrexate in the CKD population

The CKD model for MTX was adjusted based on pathological and physiological changes observed in CKD patients compared to a healthy population model, to accommodate various disease stages (Malik et al., 2020). LD-MTX is contraindicated for patients with advanced CKD (Sparks et al., 2021). We only simulated mild to moderate CKD, generating 1,000 virtual individuals randomly for each stage of CKD (stages 1–3) for the CKD population simulation. Stage 1 estimated glomerular filtration rate (eGFR) is greater than 90 (ml/min/1.73 m^2^), Stage 2 eGFR is 60–89 (ml/min/1.73 m^2^), Stage 3a eGFR is 45–59 (ml/min/1.73 m^2^), and Stage 3b eGFR is 30–44 (ml/min/1.73 m^2^) (Vaidya and Aeddula, 2025). Additionally, according to the intact nephron hypothesis (INH), the GFR change rate in INH can be used to represent the transporter change rate in mild to moderate CKD, such that eGFR in CKD patients (eGFR_CKD_) is proportional to the reference eGFR (eGFR_ref_) (Bricker, 1969; Hsueh et al., 2018). The concentration of transport proteins in the CKD virtual population is adjusted according to Equation 1

$$\text{Transporter concentration ratio}=\frac{{\text{eGFR}}_{\text{CKD}}}{{\text{eGFR}}_{\text{ref}}}$$ (1)

The eGFR_ref_ value is set to 106.78 (ml/min/1.73 m^2^), which was obtained using PK-Sim^®^ based on the European population (ICRP, 2002) with an average age of 30 years, height of 176 cm, and weight of 73 kg for a virtual healthy male.

### 2.5 PBPK model evaluation

The evaluation of the PBPK models was conducted using standard metrics, including the average mean relative deviation (MRD) (Equation 2) and the geometric mean fold error (GMFE) (Equation 3), to assess overall model accuracy. Data analysis was conducted using R version 4.4.1 (R Institute for Statistical Computing, Vienna, Austria). MRD was employed to assess the accuracy of plasma concentration predictions, while GMFE was utilized to evaluate the precision of first to the last data point (AUC_last_), and maximum observed plasma concentration (C_max_)estimates. When MRD and GMFE ≤2, model performance was considered acceptable. Furthermore, goodness-of-fit (GOF) plots were generated to visually compare the predicted concentration-time curves with the observed data from clinical studies, thereby evaluating the consistency between predicted and observed values. These primarily included plasma concentration, C_max,_ and AUC_last_ (Britz et al., 2020).

$$\text{MRD}=10^{x};\;x=\sqrt{\frac{\sum_{\;i=1}^{\;m}{\;\left(\log_{10}C_{\text{predicted},i\;}‐\;\log_{10}C_{\text{observed},i}\right)}^{2}}{m}}$$ (2)

Where C_Predicted, i_ is the predicted plasma concentration, C_observed, i_ is the corresponding observed plasma concentration, and m is the number of observations.

$$\text{GMFE}=10^{x};\;x=\frac{\sum_{\;i=1}^{\;n}\left|\log_{10}\left(\frac{{\text{predicted}\;\text{PK}\;\text{parameter}}_{i}}{{\text{observed}\;\text{PK}\;\text{parameter}}_{i}}\right)\right|}{n}$$ (3)

Where predicted PK parameteri is the predicted AUC_last_ or C_max_, observed PK parameteri is the corresponding observed AUC_last_ or C_max_, and n is the number of studies.

### 2.6 Risk threshold for adverse reactions in CKD patients

Research on the risk threshold for related toxicity in CKD patients receiving LD-MTX is currently lacking. According to Yang et al. (2018), renal function is correlated with MTX concentration, and elevated MTX levels can serve as predictors of MTX-related toxicity. Therefore, we hypothesized whether LD-MTX could be used to predict the risk of related toxicity. A study on LD-MTX use in individuals with normal renal function identified a C_max_ of 0.16 μmol/L after the first weekly dose as the threshold for MTX-related adverse reactions, with MTX dosage being positively correlated with C_max_ (Shoda et al., 2007). However, this value represents a statistical threshold and does not provide the corresponding baseline dose. Given the potential nonlinear relationship between C_max_ and dose, we considered using the RCS model to explore this relationship. The RCS model is suitable for this purpose because it allows flexible modeling of complex associations between continuous variables without a predefined functional form (Desquilbet and François, 2010), which enables us to infer the benchmark dose corresponding to 0.16 μmol/L. The RCS model was modified based on the case provided by Discacciati et al. (2025). Since C_max_ and dose are both continuous variables, ordinary least squares were used to set the RCS as a linear regression model, with the number of RCS knots defined as four and the knot locations using the default settings. The simulated data for RCS were obtained by further simulating and expanding existing healthy population data (Supplementary Table S7) with a normal distribution. The model sensitivity was assessed using the Akaike Information Criterion, and the nonlinear relationship between C_max_ and dose M was obtained through the RCS model (Equation 4). Detailed related parameters are provided in the supplementary material

$$C_{\max}=\beta_{0}+\beta_{1}M+\beta_{2}\cdot f_{1}\left(M\right)+\beta_{3}\cdot f_{2}\left(M\right)$$ (4)

Where β_0_ = 0.0636; β_1_ = 0.0139; β_2_ = 0.0542; β_3_ = −0.0912. M represents the administered dose. The nonlinear basis functions f_1_ and f_2_ (Equations 5, 6) are defined as:

$$f_{1={\left(M‐k_{1}\right)}^{3}‐\left[\left(\frac{k_{4}‐k_{1}}{k_{4}‐k_{3}}\right){\cdot \left(M‐k_{3}\right)}^{3}\right]+\left[\left(\frac{k_{3}‐k_{1}}{k_{4}‐k_{3}}\right){\cdot \left(M‐k_{4}\right)}^{3}\right]}$$ (5)

$$f_{2={\left(M‐k_{2}\right)}^{3}‐\left[\left(\frac{k_{4}‐k_{2}}{k_{4}‐k_{3}}\right){\cdot \left(M‐k_{3}\right)}^{3}\right]+\left[\left(\frac{k_{3}‐k_{2}}{k_{4}‐k_{3}}\right){\cdot \left(M‐k_{4}\right)}^{3}\right]}$$ (6)

Where k represents the knot position, which is placed at the 0.05, 0.275, 0.725, and 0.95 percentiles of the dose distribution by default, k_1_ = 5; k_2_ = 7.5; k_3_ = 15; k_4_ = 22.5.

According to Equation 4, the reference dose corresponding to a C_max_ of 0.16 μmol/L was determined. Subsequently, the established PBPK model for healthy individuals was employed to verify whether this reference dose yielded simulation outputs within the expected deviation range of 0.16 μmol/L. By integrating the RCS model outputs with the verification results from the PBPK model, the final reference dose (m_0_) was identified as 2.3 mg. On this basis, we further extrapolated the toxicity threshold by proportionally scaling the 0.16 μmol/L cutoff according to the dose ratio. In parallel, the degree of renal impairment in the CKD population was incorporated by adjusting for the ratio of GFR in CKD patients relative to that in healthy individuals. The final adjustment equation for the CKD risk threshold is presented in Equation 7.

$$C_{\max,R,T}\left(\text{Risk Thresholds}\right)=0.16\times \frac{M}{m_{0}}\times \frac{{\text{eGFR}}_{\text{CKD}}}{{\text{eGFR}}_{\text{ref}}}$$ (7)

Where M represents the administered dose, m_0_ denotes the baseline dose, eGFR_CKD_ denotes the eGFR values for each stage of CKD, and eGFR_ref_ equals 106.78, which represents the reference value for eGFR in healthy individuals. This value was obtained using PK-Sim^®^ based on a virtual healthy male with an average age of 30 years, height of 176 cm, and weight of 73 kg, derived from the European population (ICRP, 2002).

## 3 Results

### 3.1 Characteristics of methotrexate adverse events in patients with renal impairment

Between Q1 2004 and Q3 2024, 1,529,492 AEs in patients with renal impairment were identified following deduplication, including 2,663 cases associated with MTX. Table 1 presents the clinical baseline characteristics of the 2,663 cases. The gender distribution was 29.29% male and 53.59% female, with 45.59% of patients aged 65 years or older. Regarding MTX dosage, 59.63% of cases had unknown dosages, followed by the most frequent specified dosages: 2.5 mg (11.87%), 10 mg (7.55%), 15 mg (4.96%), and 20 mg (5.33%). The majority of reports originated from Canada (37.36%), the United States (19.79%), and Spain (11.34%), with 65.04% of cases reported between 2019 and 2024.

**TABLE 1:** Characteristics of patients with renal impairment experiencing adverse events associated with methotrexate (n^a^ = 2,663).

Class	Case (n)	Percent (%)
Sex		
Female	1,427	53.59
Male	780	29.29
Missing or unknown	456	17.12
Age		
0–18	198	7.44
19–45	130	4.88
45–65	641	24.07
65–100	1,214	45.59
Missing or unknown	480	18.02
Methotrexate dose		
2.5 mg	316	11.87
10 mg	201	7.55
15 mg	132	4.96
20 mg	142	5.33
Other doses	284	10.66
Missing or unknown	1,588	59.63
Reporter		
Physician	1,091	40.97
Consumer	648	24.33
Health professional	636	23.88
Other reporter	208	7.81
Missing or unknown	80	3.00
Reporting country		
CA^b^	995	37.36
US^c^	527	19.79
ES^d^	302	11.34
Other country	796	29.90
Missing or unknown	43	1.61
Report years		
2019–2024	1732	65.04
2013–2018	923	34.67
2004–2012	8	0.29

^a^ n, number.

^b^ CA, Canada.

^c^ US, United States.

^d^ ES, spain.

### 3.2 Pharmacovigilance analysis of methotrexate in patients with renal impairment

**FIGURE 3:**
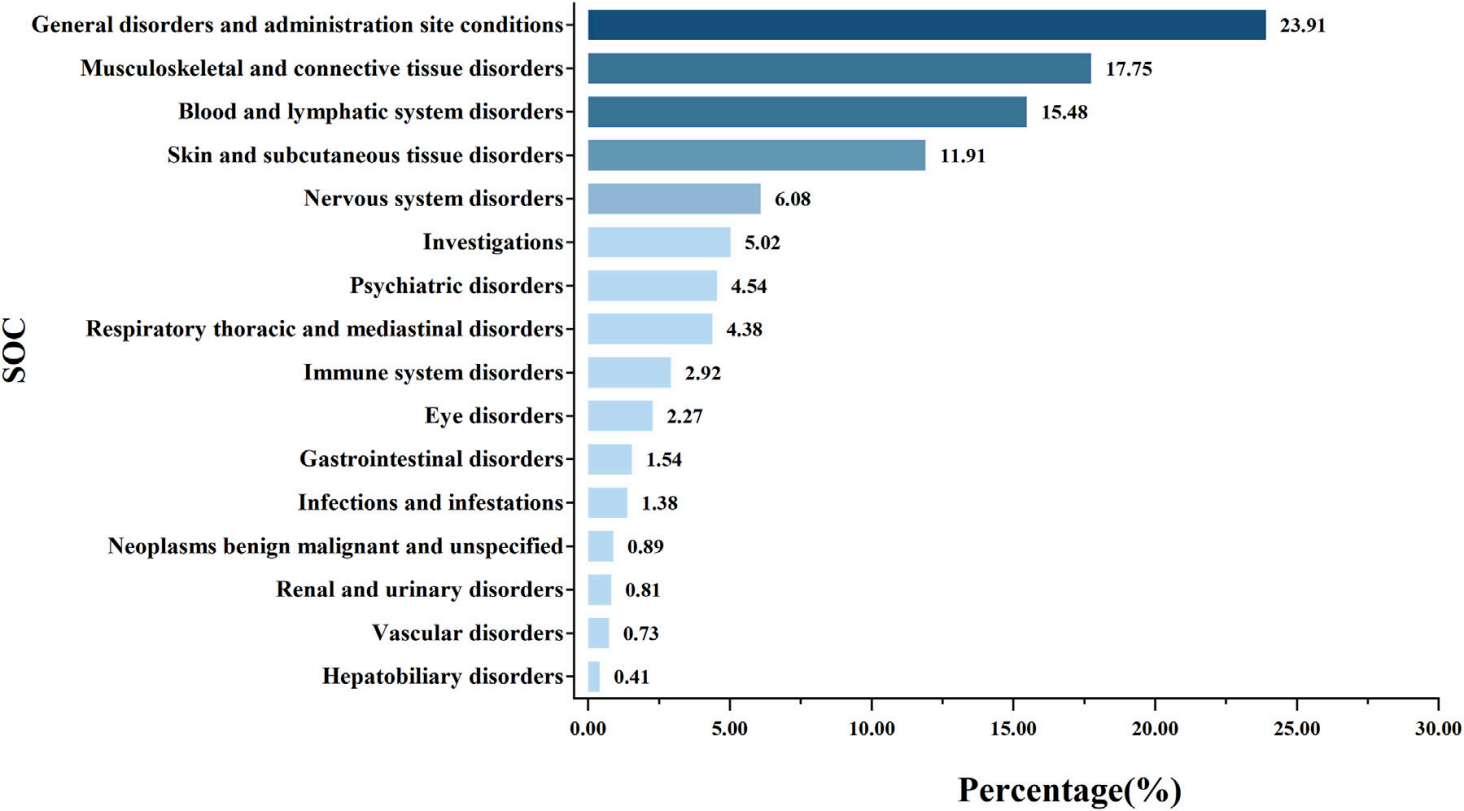
Proportion of systemic adverse events in renal impairment patients following methotrexate use.

Further analysis using PRR, ROR, and BCPNN identified a total of 75 AE signals. Based on MedDRA, these 75 signals were classified using SOC, identifying 16 SOCs for all AE signals. As illustrated in Figure 3, the SOCs associated with AE signals in the renal failure population primarily focused on general disorders and administration site conditions (23.91%), musculoskeletal and connective disorders (17.75%), blood and lymphatic system disorders (15.48%), and skin and subcutaneous tissue disorders (11.91%).

**FIGURE 4:**
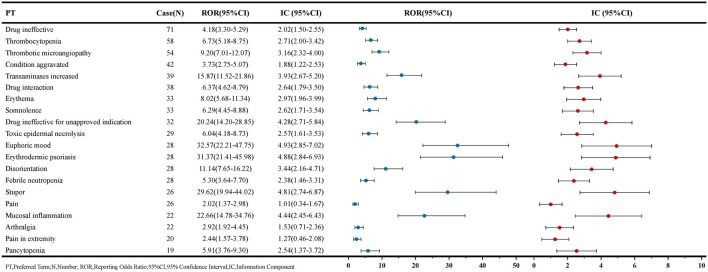
PT signal results based on the top 20 AEs reported in MTX reports.

The PT signals associated with MTX in patients with renal impairment are listed in Figure 4, ordered by the number of reports. The AEs most commonly associated with MTX include hematological disorders, liver function impairment, and skin disorders. Among these, thrombocytopenia [58 reports, ROR: 6.73, 95% CI: 5.18–8.75], pancytopenia [19 reports, ROR: 5.91, 95% CI: 3.76–9.30], and febrile neutropenia [28 reports, ROR: 5.30, 95% CI: 3.64–7.70] are associated with bone marrow suppression, while transaminases increased [39 reports, ROR: 15.87, 95% CI: 11.52–21.86] are one of the factors contributing to hepatic dysfunction. Additionally, some AEs not mentioned in the MTX package insert but related to skin diseases, such as toxic epidermal necrolysis [29 reports, ROR: 6.04, 95% CI: 4.18–8.73] and erythrodermic psoriasis [28 reports, ROR: 31.37, 95% CI: 21.41–45.98], may represent potential new AE signals. AE signals with high signal strength are primarily pulmonary and joint-related adverse reactions, with pulmonary adverse reaction signals such as rheumatoid lung [12 reports, ROR: 49.72, 95% CI: 27.53–89.77] and pulmonary toxicity [12 reports, ROR: 47.33, 95% CI: 26.24–85.37]. Joint-related adverse reaction signals include hand deformity [12 reports, ROR: 49.36, 95% CI: 27.34–89.12], tenosynovitis [12 reports, ROR: 42.14, 95% CI: 23.42–75.82], and synovitis [14 reports, ROR: 34.78, 95% CI: 20.25–59.72]. Furthermore, AEs with a certain signal strength but not mentioned in the MTX package insert include euphoric mood, B-cell lymphoma, and dry eyes. The complete signal results and signal strength ranking results are available in Supplementary Tables S1 and Supplementary Figure S1.

### 3.3 Development and validation of PBPK model for methotrexate in healthy individuals

**FIGURE 5:**
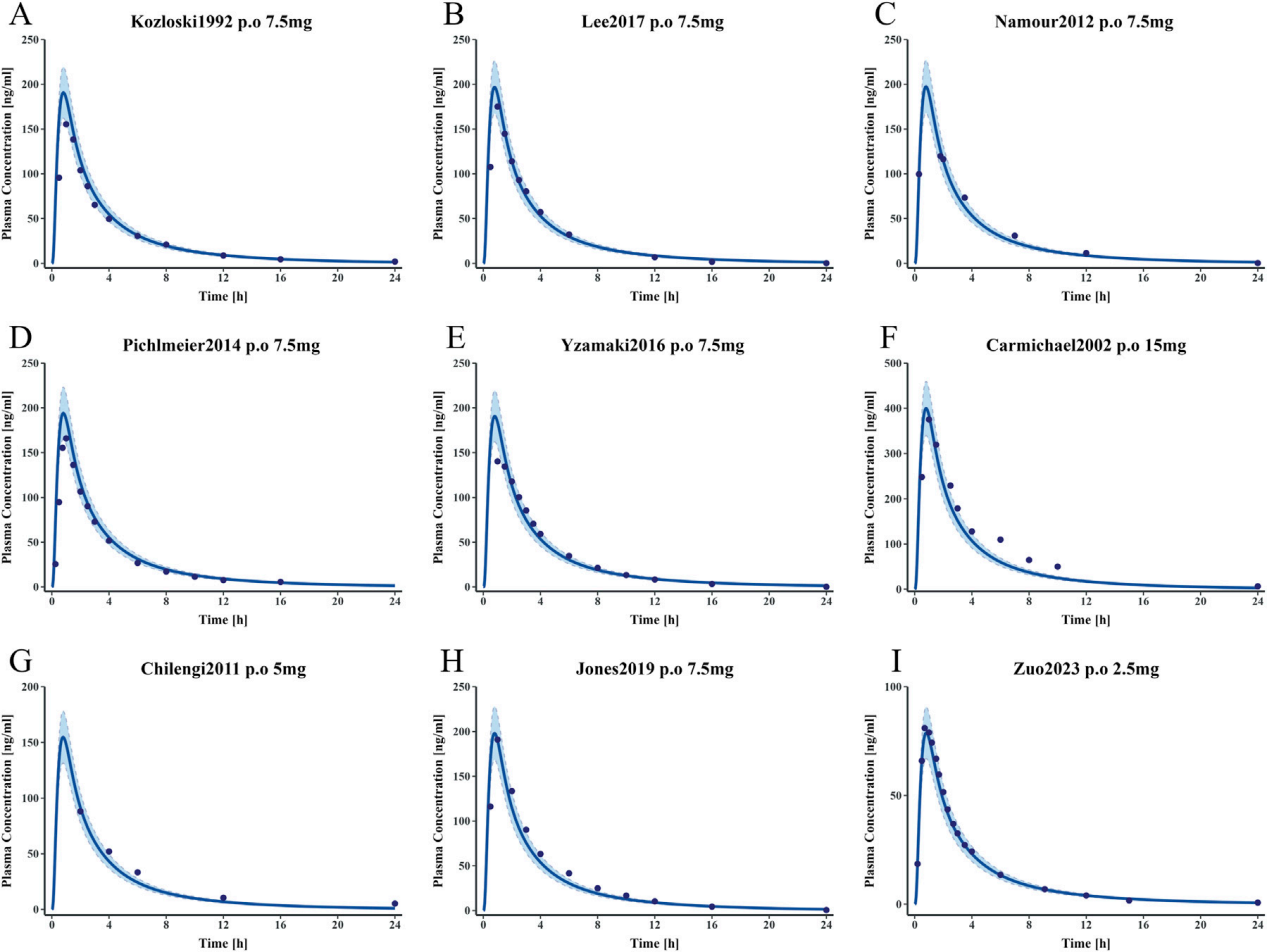
Simulated and observed plasma concentration-time curves of the methotrexate PBPK model in healthy adults **(A–E)** represent plasma concentration-time curves for the training dataset. **(F–I)** represent plasma concentration-time curves for the test dataset. Clinically observed data are shown as mean values (dark blue solid points); the dark blue solid line indicates predicted plasma concentrations; the blue shaded area represents the 95% confidence interval.

The methotrexate PBPK model for healthy individuals was constructed using different doses and time data for healthy individuals. Figure 5 presents the MTX plasma concentration-time profile. This model can accurately describe the plasma concentration distribution of MTX in healthy individuals at different doses. Supplementary Tables S2 in the supplementary materials provides complete clinical data information for training and testing. Supplementary Tables S3 provides the detailed parameters input into the model, and the results of the parameter sensitivity analysis are detailed in Supplementary Figure S2.

**FIGURE 6:**
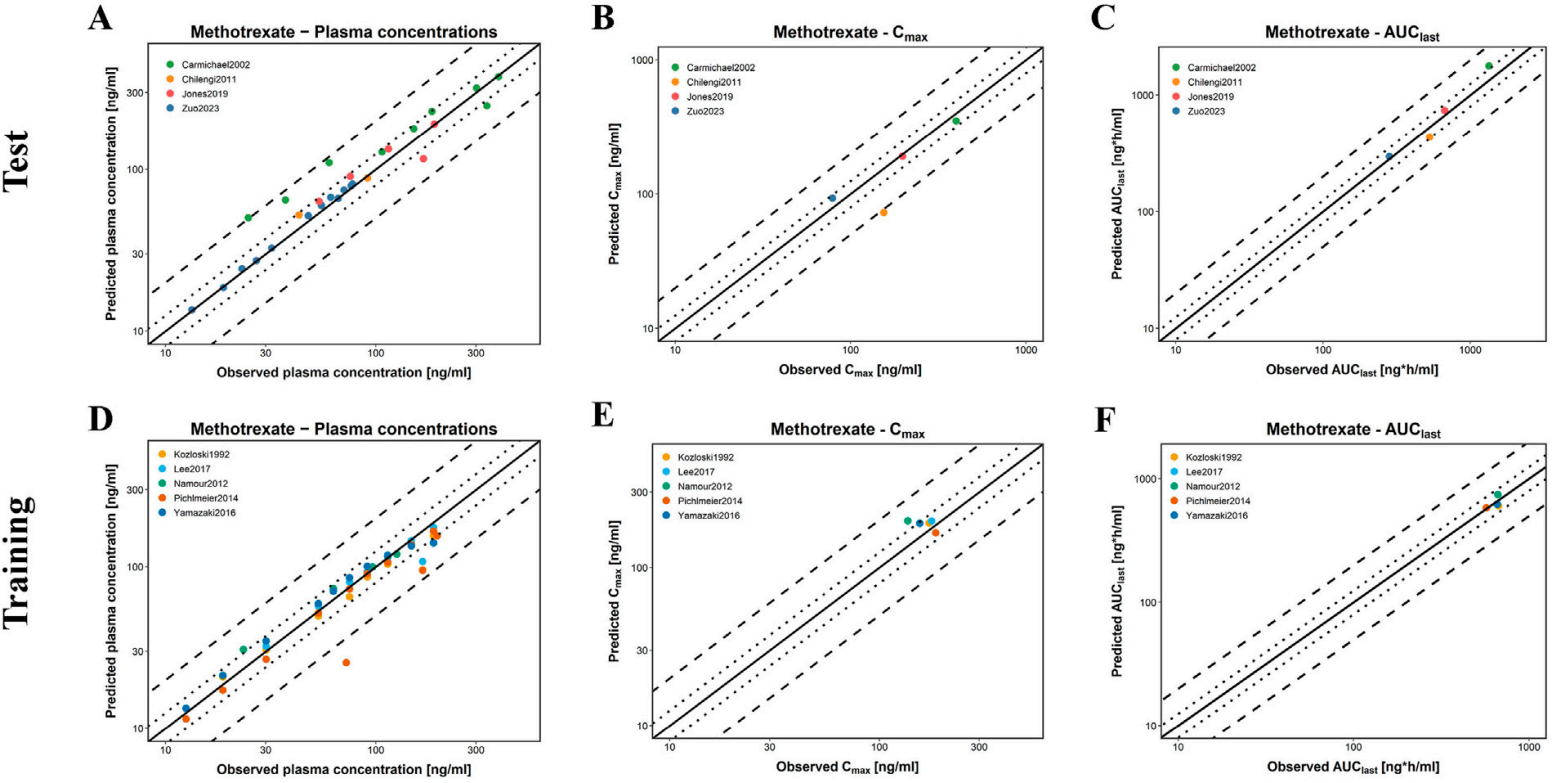
Evaluation of model performance for MTX in healthy individuals. **(A–C)** are the test groups; **(D–F)** are the training groups; **(A,D)** represent the predicted and observed MTX plasma concentrations for all clinical studies; **(B,E)** represent the predicted and observed C_max_ of MTX; **(C,F)** represent the predicted and observed AUC_last_ of MTX; solid lines indicate the identity line; dotted lines indicate a 1.25-fold deviation; dashed lines indicate a 2-fold deviation.

Figure 6 presents GOF plots for predicted *versus* observed plasma concentrations, Area under the concentration curve from the AUC_last_, and C_max_ in the training and test groups. The predicted plasma concentration values (98.04%), C_max_ (9/9), and AUC_last_ (9/9) fall within a two-fold range of the observed data. Regarding model performance, the average MRD value for this model is 1.50, with GMFE values of 0.99 and 1.04 for AUC_last_ and C_max_, respectively. All these values are less than 2, indicating good model performance. Detailed parameters are available in Supplementary Tables S4–6.

### 3.4 Risks of low-dose methotrexate in individuals with CKD

**FIGURE 7:**
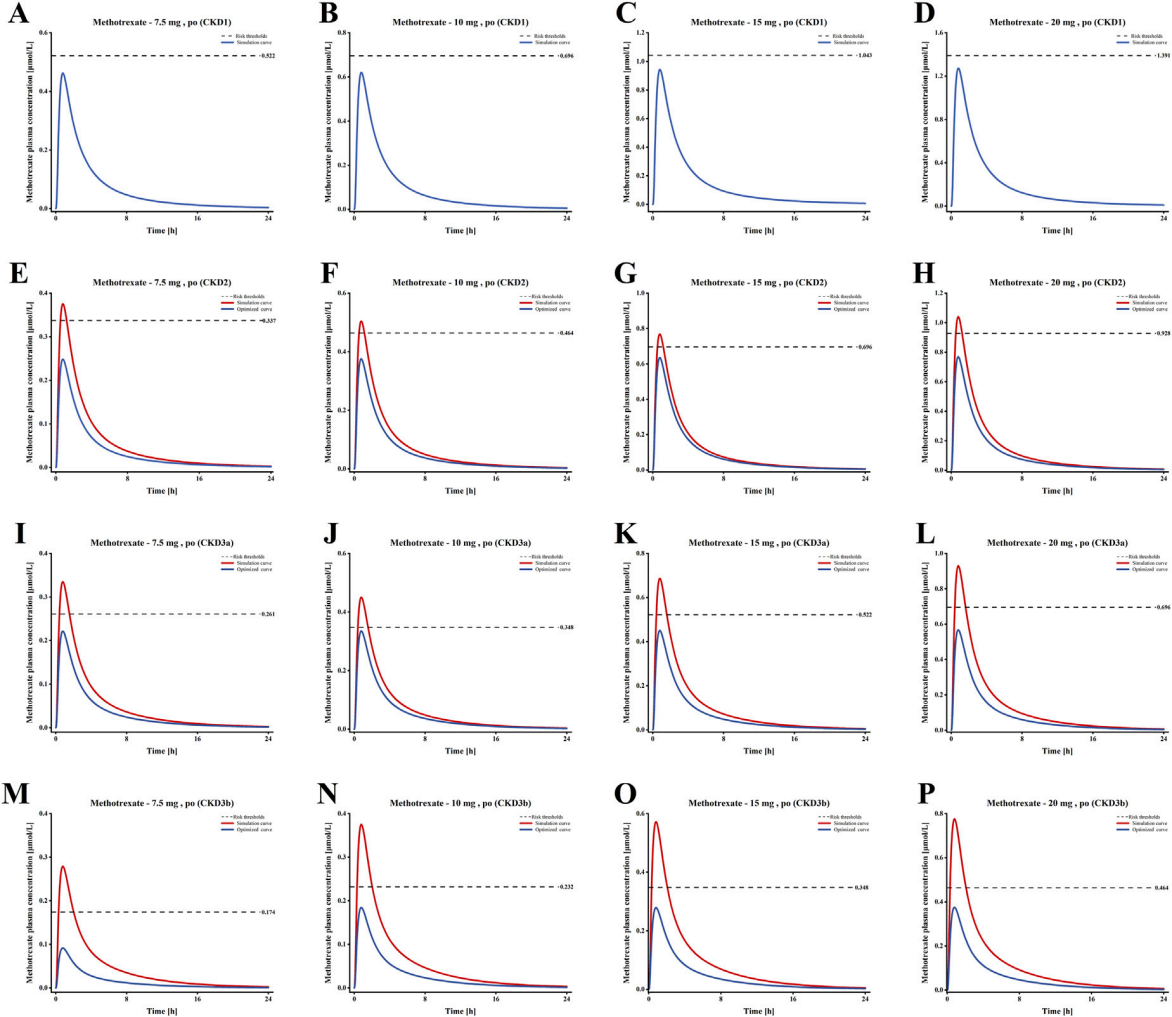
Plasma concentration time profiles of MTX in populations with different Stages of CKD. **(A–D)** Plasma concentration-time profiles under standard dosing regimens for Stage 1 CKD; **(E–H)** Plasma concentration-time profiles under standard dosing regimens and optimized dosing regimens for Stage 2 CKD; **(I–L)** Plasma concentration-time profiles under standard dosing regimens and optimized dosing regimens for Stage 3a CKD; **(M–P)** Plasma concentration-time profiles under standard dosing regimens and optimized dosing regimens for Stage 3b CKD.

According to the FDA drug label, the LD-MTX dosing regimens are as follows: 1) 7.5 mg once weekly tablet po; 2) 10 mg once weekly tablet po; 3) 15 mg once weekly tablet po; 4) 20 mg once weekly tablet po. Table 2 shows the plasma risk thresholds for individuals with mild to moderate CKD under standard oral dosing regimens. Figure 7 illustrates the plasma concentration-time profile of MTX in individuals with mild to moderate CKD. Simulation results for each stage of CKD indicate that in Stage 1 CKD, plasma concentrations for all four oral dosing regimens remained below the predicted plasma risk threshold. In Stages 2 through 3b of CKD, plasma concentrations for all four dosing regimens exceeded the predicted plasma risk threshold. Additionally, starting from Stage 3a CKD, the plasma concentrations simulated by the PBPK model significantly exceeded the predicted plasma risk threshold, with this phenomenon becoming more pronounced in Stage 3b CKD. These findings suggest that, beginning with Stage 2 CKD, there is a potential risk of adverse reactions associated with the use of LD-MTX in patients with mild to moderate renal impairment.

**TABLE 2:** Maximum plasma concentration risk thresholds for common oral medication regimens in people with mild to moderate CKD.

CKD [stage]	eGFR [ml/min/1.73m^2^]	7.5 mg C_max,R,T_ [μmol/L]	10 mg C_max,R,T_ [μmol/L]	15 mg C_max,R,T_ [μmol/L]	20 mg C_max,R,T_ [μmol/L]
CKD1	90	0.522	0.696	1.043	1.391
CKD2	89	0.516	0.688	1.032	1.377
	60	0.348	0.464	0.696	0.928
CKD3a	59	0.342	0.456	0.684	0.912
	45	0.261	0.348	0.522	0.696
CKD3b	44	0.255	0.340	0.510	0.681
	30	0.174	0.232	0.348	0.464

C_max,R,T_: Risk Thresholds C_max_.

### 3.5 Optimization of low-dose methotrexate regimens for patients with CKD

Based on the simulation results shown in Figure 7, we optimized the MTX dosage for patients with CKD Stages 2–3b to reduce the risk of adverse reactions. The standard tablet dosage of 2.5 mg, as specified in the drug label, served as the baseline for these optimizations. The blue curve of E-P in Figure 7 shows the optimized blood drug concentration results, Table 3 presents the optimized oral dosage regimen, with the specific optimization details as follows: For patients with CKD Stage 2, the initial dosage was reduced by 2.5 mg (one tablet) to ensure that the maximum plasma concentration remained below the risk threshold; For patients with CKD Stage 3a, the initial regimens of 7.5 mg and 10 mg were adjusted to 5 mg and 7.5 mg, while the 15 mg and 20 mg regimens were modified to 10 mg and 12.5 mg; Similarly, for patients with CKD Stage 3b, the initial 7.5 mg and 10 mg regimens were reduced to 2.5 mg and 5 mg, and the 15 mg and 20 mg regimens to 7.5 mg and 10 mg, to maintain the peak plasma concentration below the risk threshold.

**TABLE 3:** Optimal dosage regimen for methotrexate in CKD.

CKD [stage]	Initial dose (mg)	Optimized dose (mg)	Dose reduction (mg/tablets)
CKD2	7.5	5	2.5 mg (1 Tablet)
	10	7.5	
	15	12.5	
	20	17.5	
CKD3a	7.5	5	2.5–7.5 mg (1–3 Tablets)
	10	7.5	
	15	10	
	20	12.5	
CKD3b	7.5	2.5	5–10 mg (2–4 Tablets)
	10	5	
	15	7.5	
	20	10	

## 4 Discussion

LD-MTX does not significantly exacerbate renal impairment, but impaired renal function reduces its excretion from the body, thereby increasing the risk of other adverse reactions (Sparks et al., 2021). Despite increasing research on LD-MTX in recent years, comprehensive evaluation protocols for LD-MTX in patients with renal impairment remain scarce, leaving a lack of reference information. By integrating pharmacovigilance data mining with pharmacokinetics based on a PBPK model, we have revealed the adverse reaction risks of LD-MTX in patients with renal impairment. Concurrently, we further explored the pharmacokinetic changes of MTX in renal impairment and proposed dose adjustment strategies. This provides a more precise reference for future clinical adjustments to LD-MTX oral dosage regimens.

A study conducted in 2021 indicated that MTX users with CKD may be at increased risk of hematological toxicity (Mitsuboshi, 2021). In another study of elderly CKD patients, those prescribed LD-MTX had a significantly increased risk of seeking medical attention within 90 days due to myelosuppression, sepsis, pulmonary toxicity, or hepatic toxicity (Muanda et al., 2023). Similarly, our pharmacovigilance signal results also confirmed that LD-MTX is closely associated with the risk of hematological toxicity, pulmonary toxicity, or hepatic toxicity, with renal impairment possibly playing an important role. We observed that since the Kidney Disease: Improving Global Outcomes (KDIGO) updated its clinical assessment guidelines for CKD in 2012 (Andrassy, 2013), there has been a significant increase in reports of adverse reactions to MTX in patients with renal impairment, notably accounting for two-thirds of all cases reported in the last 5 years. Therefore, many patients with MTX adverse reactions before 2012 may have had some degree of renal impairment that went undiagnosed. At the same time, the increase in reports in the past 5 years also reflects that many medical professionals have begun to pay attention to the adverse reactions of LD-MTX in patients with renal impairment. Further analysis of the population characteristics report for MTX revealed that the proportion of unknown doses in the MTX dose report once reached 60%. However, we must consider the inherent limitations of spontaneous reporting system studies. The assessment of MTX in patients with renal impairment usually requires dose adjustment based on the degree of renal impairment. However, the lack of key dose information in FAERS limits the evaluation of the relationship between dose and renal impairment. At the same time, cases of renal impairment identified in adverse reaction reports through diagnostic codes such as MedDRA high-level terms often lack accurate information on the stage of renal impairment, which makes it difficult to determine the relationship between the degree of renal impairment and adverse reactions. In addition, the reliability of case characteristic information is highly dependent on the completeness of the reporter’s records, and the missing dose information observed in this study further emphasizes the importance of completeness of records. Although many reports are submitted by professionals such as medical workers, given the spontaneous nature of the data, the possibility of incomplete or even misidentified information must be taken into account. Finally, even though adverse reaction signals in FAERS can point to potential risk concerns, there’s no corresponding control group data for adverse reactions, so these results need to be explored through rigorously designed confirmatory research to verify the relationship between actual clinical risk characteristics and related factors.

In recent years, while a comprehensive monitoring system exists for high-dose methotrexate adverse reactions (Howard et al., 2016), low-dose methotrexate assessment is frequently overlooked. A study has shown that the monitoring frequency for patients using LD-MTX is significantly lower than the recommended frequency (Mazaud and Fardet, 2021). Meanwhile, when patients present with multiple clinical risk factors, particularly renal impairment, regular monitoring of MTX blood levels is essential; otherwise, even LD-MTX may lead to serious adverse reactions (Yan et al., 2024). MTX is primarily excreted through the kidneys, so changes in kidney function are associated with an increased risk of MTX adverse reactions (Widemann and Adamson, 2006). PBPK models simulate the pharmacokinetic changes of MTX in patients with renal impairment to adjust dosage regimens and reduce adverse reaction risks.Although Wang et al. also developed a PBPK model for MTX use in rheumatoid arthritis patients (Wang et al., 2025), this study did not elaborate on the role of MTX transporters in renal metabolism. Furthermore, studies indicate that adverse reactions to MTX primarily stem from the parent drug rather than its metabolites (Hamed et al., 2025). This is further supported by Buchen et al.'s research, which demonstrated that carboxypeptidase G2 alleviates MTX parent drug toxicity in patients with renal impairment (Buchen et al., 2005). Therefore, elucidating the renal transport mechanisms of MTX is particularly crucial. This study provides a detailed description of the renal transport mechanisms of MTX, which served as the foundation for successfully establishing a PBPK model for MTX. Meanwhile, both the MRD and GMFE values were less than 2, indicating that the model possesses good predictive performance. Research indicates that the vast majority of MTX is excreted unchanged in urine, among which renal tubular secretion accounts for 60%–80% of MTX renal excretion. Through organic anion transporters (OAT3), reduced folate carrier (RFC1) and multidrug resistance-associated proteins (BCRP, MRP4) transport MTX from the blood to the renal tubule lumen. At the same time, renal tubule function plays an important role in the excretion of endogenous substances in CKD patients (Risso et al., 2019; Christophidis et al., 1981). We adjusted the concentrations of tubular-associated proteins based on the INH hypothesis, while incorporating partial hepatic metabolism and biliary excretion to simulate non-renal MTX clearance. Based on a healthy individual model, we supplemented the investigation of dosage risks associated with MTX use in CKD populations by simulating MTX dosing regimens for CKD patients at different stages. Although individuals in different populations may respond differently to LD-MTX, determining a reasonable risk threshold is crucial. Although Shoda et al. inferred that 0.16 μmol/L is the critical concentration for increased adverse reactions to MTX in individuals with normal renal function (Shoda et al., 2007), this threshold is a statistical threshold and cannot be used as a clinical diagnostic criterion. Therefore, defining the risk threshold for LD-MTX remains a significant challenge. Given that KDIGO recommends adjusting the dose of renally excreted drugs based on eGFR, and since MTX primarily relies on renal clearance, we attempted to combine this threshold, drug dose, and CKD stage. We used the RCS model to derive the nonlinear relationship between MTX C_max_ and dose (Desquilbet and François, 2010), reverse-engineered the safe baseline dose of 0.16 μmol/L, and then scaled up this threshold by setting a dose ratio based on the baseline dose. We hypothetically set the dynamic risk threshold for CKD (Equation 7), and this threshold was reasonably optimized and adjusted in the PBPK model.The FDA recommends reducing or discontinuing MTX in patients with renal impairment and monitoring blood drug concentrations. However, there is no clear guidance on how to reduce the dose or discontinue the medication. The PBPK simulation results in this study indicate that, starting from stage 2 CKD, the use of LD-MTX at conventional doses carries a certain risk of adverse reactions. Previous reports on adverse reactions caused by LD-MTX in the CKD population support these simulation results (Muanda et al., 2023; Sparks et al., 2021). Additionally, we modified dosage regimens for CKD stages 2–3b using risk thresholds and the PBPK model to mitigate adverse reaction risks. Nevertheless, in practical application, the CKD model in this research still has some aspects that require further attention. Firstly, the model is constructed based on theoretical deduction and has certain theoretical guidance significance. But due to the current lack of sufficient clinical data to comprehensively verify its applicability in the CKD population, we are currently unable to conduct a complete empirical evaluation. Second, the activity and expression levels of transporters may vary significantly among individuals and disease states. For specific transporters (RFC1, MRP4), assessing non-linear declines across CKD stages and accurately simulating MTX pharmacokinetics remain challenging. Therefore, while PBPK models can provide quantitative predictions, their outcomes depend on assumptions and parameters, requiring further experimental and clinical validation. In summary, the pharmacovigilance-PBPK combined analysis in this study indicates that LD-MTX carries a risk of adverse reactions in patients with mild to moderate renal impairment. Blood monitoring and dose adjustment are recommended during its use.

## 5 Conclusion

In this study, analysis of the FAERS database first indicated that low-dose methotrexate use in patients with renal impairment may lead to hematologic disorders, hepatic impairment, and pulmonary and joint-related adverse reactions. Secondly, the established PBPK model successfully predicted plasma concentration distribution of methotrexate in healthy individuals and further supplemented dose-risk assessment for methotrexate use in CKD populations. PBPK simulation results indicate that conventional oral dosing regimens of methotrexate carry a certain risk of adverse reactions starting from stage 2 CKD. Oral dose optimization based on the drug label is recommended, with the following specific optimization schemes: For CKD stage 2, reduce the initial dose by 2.5 mg across all initial dosing regimens; For CKD stage 3a, the initial 7.5 mg and 10 mg regimens are adjusted to 5 mg and 7.5 mg, and the initial 15 mg and 20 mg regimens are adjusted to 10 mg and 12.5 mg; For CKD stage 3b, the initial 7.5 mg and 10 mg regimens are adjusted to 2.5 mg and 5 mg, and the initial 15 mg and 20 mg regimens are adjusted to 7.5 mg and 10 mg.

## Data Availability

The original contributions presented in the study are included in the article/Supplementary Material, further inquiries can be directed to the corresponding author.
