# Supplementary material for: Low-dose methotrexate adverse reaction risk in renal impairment: pharmacovigilance and physiological pharmacokinetic model assessment
Source: Front Pharmacol. 2025 Nov 6;16:1703557. doi: 10.3389/fphar.2025.1703557 (PMC12631143; doi:10.3389/fphar.2025.1703557)
Supplement: Supplementary file 1 [file Supplementaryfile1.pdf]

Table S1 . Complete PT signal information for MTX in patients with renal impairment

SOC	PT	Case(n)	ROR(95%CI)	PRR(χ2)	IC(IC025)
**General disorders and administration site conditions**	Drug ineffective	71	4.18(3.30-5.29)	4.09(165.69)	2.02(1.50)
	Condition aggravated	42	3.73(2.75-5.07)	3.69(82.19)	1.88(1.22)
	Drug interaction	38	6.37(4.62-8.79)	6.30(167.86)	2.64(1.79)
	Drug ineffective for unapproved indication	32	20.24(14.20-28.85)	20.01(558.71)	4.28(2.71)
	Pain	26	2.02(1.37-2.98)	2.01(13.29)	1.01(0.34)
	Mucosal inflammation	22	22.66(14.78-34.76)	22.48(434.76)	4.44(2.45)
	Feeling abnormal	14	3.22(1.90-5.46)	3.21(21.24)	1.68(0.60)
	Multiple organ dysfunction syndrome	13	2.69(1.56-4.65)	2.68(13.69)	1.42(0.38)
	Treatment failure	13	14.37(8.28-24.95)	14.31(157.03)	3.81(1.70)
	Drug intolerance	12	9.35(5.28-16.56)	9.31(87.63)	3.20(1.39)
	Illness	12	9.22(5.21-16.33)	9.19(86.20)	3.18(1.38)
**Musculoskeletal and connective tissue disorders**	Arthralgia	22	2.92(1.92-4.45)	2.90(27.38)	1.53(0.71)
	Pain in extremity	20	2.44(1.57-3.78)	2.42(16.72)	1.27(0.46)
	Joint swelling	18	5.04(3.16-8.02)	5.01(57.35)	2.31(1.19)
	Joint range of motion decreased	16	30.66(18.51-50.76)	30.48(433.22)	4.86(2.17)
	Myalgia	15	4.01(2.41-6.67)	3.99(33.44)	1.99(0.86)
	Musculoskeletal stiffness	15	9.48(5.68-15.81)	9.43(111.28)	3.22(1.59)
	Synovitis	14	34.78(20.25-59.72)	34.60(430.86)	5.03(1.99)
	Fibromyalgia	13	20.18(11.59-35.14)	20.09(227.90)	4.28(1.82)
	Synovial cyst	12	22.88(12.83-40.79)	22.78(240.41)	4.46(1.75)
	Hand deformity	12	49.36(27.34-89.12)	49.14(521.34)	5.50(1.65)
	Muscular weakness	12	3.23(1.83-5.71)	3.22(18.30)	1.68(0.52)

	Tenosynovitis	12	42.14(23.42-75.82)	41.95(447.06)	5.29(1.71)
	Mobility decreased	12	3.94(2.23-6.96)	3.93(26.06)	1.97(0.71)
	Rheumatoid arthritis	9	12.12(6.26-23.48)	12.08(89.64)	3.57(1.22)
	Psoriatic arthropathy	6	22.83(10.09-51.67)	22.78(120.19)	4.46(0.62)
	Musculoskeletal pain	6	4.60(2.06-10.28)	4.59(16.74)	2.19(0.30)
	Osteonecrosis	5	7.05(2.92-17.06)	7.04(25.62)	2.80(0.33)
**Blood and lymphatic system disorders**	Thrombocytopenia	58	6.73(5.18-8.75)	6.61(273.91)	2.71(2.00)
	Thrombotic microangiopathy	54	9.20(7.01-12.07)	9.04(380.85)	3.16(2.32)
	Febrile neutropenia	28	5.30(3.64-7.70)	5.25(95.74)	2.38(1.46)
	Pancytopenia	19	5.91(3.76-9.30)	5.88(76.19)	2.54(1.37)
	Neutropenia	16	3.80(2.32-6.22)	3.78(32.62)	1.91(0.84)
	Myelosuppression	8	12.33(6.11-24.87)	12.30(81.30)	3.59(1.09)
	Coagulopathy	8	4.84(2.41-9.72)	4.83(24.09)	2.26(0.59)
	Erythema	33	8.02(5.68-11.34)	7.94(197.64)	2.97(1.96)
**Skin and subcutaneous tissue disorders**	Toxic epidermal necrolysis	29	6.04(4.18-8.73)	5.99(119.45)	2.57(1.61)
	Erythrodermic psoriasis	28	31.37(21.41-45.98)	31.05(772.79)	4.88(2.84)
	Psoriasis	12	17.40(9.79-30.95)	17.33(179.29)	4.07(1.68)
	Rash maculo-papular	12	8.00(4.52-14.17)	7.97(72.21)	2.98(1.29)
	Rash pruritic	12	8.22(4.65-14.56)	8.19(74.73)	3.02(1.31)
	Dry skin	6	6.03(2.69-13.48)	6.01(24.83)	2.58(0.46)
	Skin burning sensation	5	11.77(4.85-28.55)	11.75(48.19)	3.53(0.42)
	Skin plaque	5	20.37(8.34-49.74)	20.33(88.76)	4.30(0.31)
	Papule	5	11.00(4.54-26.68)	10.98(44.53)	3.43(0.42)
**Nervous system disorders**	Somnolence	33	6.29(4.45-8.88)	6.22(143.29)	2.62(1.71)
	Stupor	26	29.62(19.94-44.02)	29.34(677.42)	4.81(2.74)

	Status epilepticus	7	8.13(3.85-17.15)	8.11(43.03)	3.00(0.78)
	Hyperaesthesia	5	20.22(8.28-49.38)	20.19(88.10)	4.29(0.32)
	Neurotoxicity	4	11.37(4.22-30.61)	11.35(37.04)	3.48(0.06)
**Investigations**	Transaminases increased	39	15.87(11.52-21.86)	15.65(521.05)	3.93(2.67)
	Bone density decreased	15	10.00(6.00-16.69)	9.95(118.82)	3.29(1.63)
	Hepatic enzyme increased	8	6.46(3.21-12.98)	6.44(36.39)	2.67(0.79)
**Psychiatric disorders**	Disorientation	28	11.14(7.65-16.22)	11.03(250.90)	3.44(2.16)
	Euphoric mood	28	32.57(22.21-47.75)	32.24(802.65)	4.93(2.85)
**Respiratory thoracic and mediastinal disorders**	Chronic obstructive pulmonary disease	13	4.77(2.76-8.24)	4.75(38.22)	2.24(0.94)
	Rheumatoid lung	12	49.72(27.53-89.77)	49.50(524.95)	5.51(1.65)
	Pulmonary toxicity	12	47.33(26.24-85.37)	47.12(500.66)	5.45(1.67)
	Pneumonitis	12	12.89(7.26-22.87)	12.84(128.15)	3.65(1.57)
	Sputum discoloured	5	16.50(6.78-40.19)	16.48(70.66)	4.00(0.39)
**Immune system disorders**	Hypersensitivity	17	4.40(2.73-7.11)	4.38(44.11)	2.12(1.02)
	Graft versus host disease	15	10.47(6.27-17.46)	10.41(125.41)	3.36(1.66)
	Hypogammaglobulinaemia	4	11.90(4.42-32.05)	11.88(39.06)	3.54(0.06)
**Eye disorders**	Cataract	12	5.19(2.94-9.18)	5.17(40.07)	2.36(0.96)
	Dry eye	12	19.57(11.00-34.84)	19.49(203.63)	4.24(1.72)
	Eye pain	4	12.55(4.66-33.82)	12.53(41.55)	3.62(0.05)
**Gastrointestinal disorders**	Stomatitis	13	7.95(4.59-13.75)	7.91(77.48)	2.97(1.35)
	Flatulence	6	6.35(2.84-14.20)	6.34(26.67)	2.65(0.49)
**Infections and infestations**	Viral upper respiratory tract infection	13	24.88(14.26-43.40)	24.76(284.22)	4.57(1.87)
	Herpes simplex	4	12.55(4.66-33.82)	12.53(41.55)	3.62(0.05)
**Neoplasms benign malignant and**	B-cell lymphoma	7	32.45(15.14-69.55)	32.37(201.43)	4.94(0.77)

**unspecified (incl cysts and polyps)**	Marrow hyperplasia	4	12.55(4.66-33.82)	12.53(41.55)	3.62(0.05)
**enal and urinary disorders**	Nephropathy toxic	10	8.77(4.69-16.39)	8.74(67.55)	3.11(1.19)
**Vascular disorders**	Shock	9	7.58(3.92-14.64)	7.56(50.54)	2.90(1.00)
**Hepatobiliary disorders**	Hypertransaminasaemia	5	15.95(6.56-38.83)	15.93(68.06)	3.96(0.39)

ROR:Reporting Odds Ratio; PRR:Proportional Reporting Ratio; IC:Information Component;SOC: System Organ Class; 95% CI: 95% Confidence Interval; χ^2:Chi-squared Test; IC025: Lower Bound of 95% CI

PT	Case(N)	ROR(95%CI)	IC (95%CI)
Rheumatoid lung	12	49.72(27.53-89.77)	5.51(1.65-9.38)
Hand deformity	12	49.36(27.34-89.12)	5.50(1.65-9.35)
Pulmonary toxicity	12	47.33(26.24-85.37)	5.45(1.67-9.23)
Tenosynovitis	12	42.14(23.42-75.82)	5.29(1.71-8.88)
Synovitis	14	34.78(20.25-59.72)	5.03(1.99-8.07)
Euphoric mood	28	32.57(22.21-47.75)	4.93(2.85-7.02)
B-cell lymphoma	7	32.45(15.14-69.55)	4.94(0.77-9.11)
Erythrodermic psoriasis	28	31.37(21.41-45.98)	4.88(2.84-6.93)
Joint range of motion decreased	16	30.66(18.51-50.76)	4.86(2.17-7.54)
Stupor	26	29.62(19.94-44.02)	4.81(2.74-6.87)
Viral upper respiratory tract infection	13	24.88(14.26-43.40)	4.57(1.87-7.28)
Synovial cyst	12	22.88(12.83-40.79)	4.46(1.75-7.17)
Psoriatic arthropathy	6	22.83(10.09-51.67)	4.46(0.62-8.29)
Mucosal inflammation	22	22.66(14.78-34.76)	4.44(2.45-6.43)
Skin plaque	5	20.37(8.34-49.74)	4.30(0.31-8.28)
Drug ineffective for unapproved indication	32	20.24(14.20-28.85)	4.28(2.71-5.84)
Hyperaesthesia	5	20.22(8.28-49.38)	4.29(0.32-8.26)
Fibromyalgia	13	20.18(11.59-35.14)	4.28(1.82-6.74)
Dry eye	12	19.57(11.00-34.84)	4.24(1.72-6.76)
Psoriasis	12	17.40(9.79-30.95)	4.07(1.68-6.47)

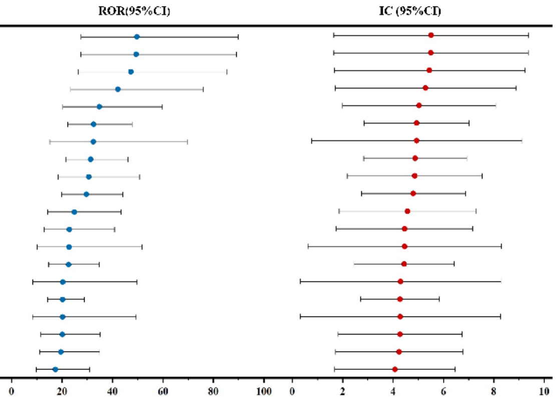

PT,Preferred Term;N,Number; ROR,Reporting Odds Ratio;95%CI,95% Confidence Interval;IC,Information Component.

Figure S1. PT signal results based on the top 20 AEs reported in MTX reports

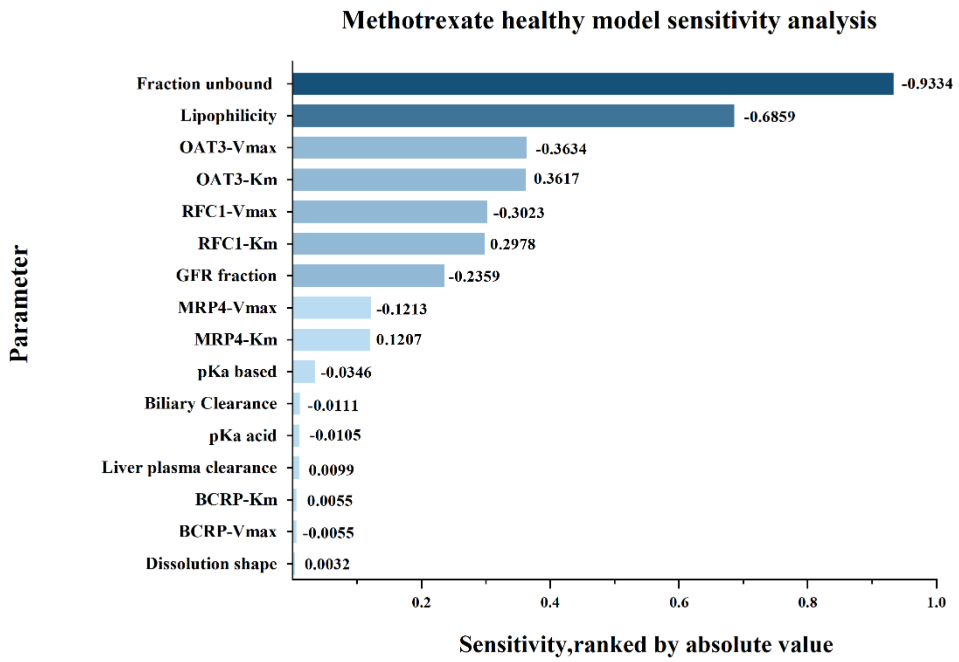

Figure S2. Sensitivity analysis of parameters in the methotrexate health PBPK model. Sensitivity to individual parameters was tested by simulating changes in AUC_{last} following oral administration of 7.5 mg. AUC_{last} refers to the area under the curve from the time of administration to the last measured concentration; K_m refers to the Michaelis-Menten constant; V_{max} refers to the maximum reaction rate; and GFR refers to the glomerular filtration rate.

Table S2. Clinical study data on methotrexate in healthy individuals

Dose[mg]	n	Female[%]	Age(SD)[years]	Weight(SD)[kg]	Height(SD)[cm]	C_{max}[mg/l]	$AUC_{0\text{-}inf}$[mg·h/l]	Dataset	References
7.5	12	-	18-45	-	-	0.173(0.040)	0.601(0.163)	Training	Kozloski1992[1]
7.5	12	-	18-50	-	-	0.178	0.625	Training	Lee2017[2]
7.5	6	-	18-50	-	-	0.137	0.744	Training	Namour2012[3]
7.5	14	21.4	47.9(5.23)	79.4(10.99)	176.1(8.24)	0.186	0.580	Training	Pichlmeier2014[4]
7.5	24	-	33.5(10.2)	80.1(12.1)	-	0.156	0.620	Training	Yamazaki2016[5]
7.5	13	-	20-55	-	-	0.191	0.736	Test	Jones2019[6]
2.5	25	48	34.04（9.21）	65.81（7.85）	168.46（7.20）	0.0929	0.295	Test	Zuo2023[7]
5	-	-	23.9(3.3)	60.78(4.92)	172.2(5.14)	0.0726	0.435	Test	Chilengi2011[8]
15	10	40	20-48	55-94	-	0.351	1.775	Test	Carmichael2002[9]

C_{max}: maximum observed plasma concentration; $AUC_{0\text{–}inf}$: area under the plasma concentration-time curve from time zero to infinity;SD: standard deviation

Table S3. Drug-dependent parameters of the methotrexate PBPK model

Parameter	Value	Unit	Source	Literature	Reference	Description
MW	454.44	g/mol	Literature	454.44	Drugbank	Molecular weight
pKa acid	3.50	-	Optimized	2.68;4.11;5.48	Alva2024[10]	Acid dissociation constant
pKa base	9.20	-	Optimized	14.55	Chemaxon	Base dissociation constant
logP	-1.54	-	Optimized	-1.85;-0.05;-2.3	Drugbank	Lipophilicity
fu	50	%	Optimized	46.5-54	Inoue2014[11]	Fraction unbound in plasma
Solubility	0.0819	mg/mL	Literature	0.0819	ALOGPS	Water Solubility
OAT3 $_{Km}$	18.50	μM	Optimized	21.10、17.2	Takeda2002[12] Uwai2004[13]	OAT3 Michaelis-Menten constant
OAT3 $_{Vmax}$	0.013	μmol/l/min	Optimized	-	-	OAT3 Maximum Velocity
BCRP $_{Km}$	690.98	μM	Optimized	680	Volk2003[14]	BCRP Michaelis-Menten constant
BCRP $_{Vmax}$	0.16	μmol/l/min	Optimized	2400 (pmol/mg/min)	Volk2003[14]	BCRP Maximum Velocity
MRP4 $_{Km}$	210	μM	Optimized	0.22±0.01(mM)	Chen2002[15]	MRP4 Michaelis-Menten constant
MRP4 $_{Vmax}$	13.50	nmol/l/min	Optimized	0.24±0.05(nmol/mg/min)	Chen2002[15]	MRP4 Maximum Velocity
RFC1 $_{Km}$	6.29	μM	Optimized	5-10	Inoue2014[11]	RFC1 Michaelis-Menten constant
RFC1 $_{Vmax}$	0.65	μmol/l/min	Optimized	-	-	RFC1 Maximum Velocity
Intestinal permeability	7.05E-6	cm/s	Literature	7.05 ± 3.64×10e-6	Michiba2021[16]-	Transcellular intestinal permeability
Tablet fasted Weibull	1.01	-	Optimized	-	-	Dissolution profile shape

shape						
Tablet fasted Weibull time	0.3	h	Optimized	1-2(Tmax)	Drugbank	Dissolution time (50% dissolved)
Liver Plasma Clearance	1.27 E-3	ml/min/kg	Optimized	-	-	Liver plasma clearance
Biliary Clearance	1.42E-3	ml/min/kg	Optimized			Biliary Clearance
GFR	1.00	-	Assumed	-	-	Glomerular Filtration Rate

OAT3:Organic Anion Transporter 3;BCRP:Breast Cancer Resistance Protein;MRP4:Multidrug Resistance Associated Protein 4;RFC1:Reduced Folate Carrier 1

Table S4. MRD values for methotrexate plasma concentrations in healthy individuals

Reference	Dose [mg]	Dataset	MRD
Kozloski1992[1]	7.5	Training	1.22
Lee2017[2]	7.5	Training	1.83
Namour2012[3]	7.5	Training	1.69
Pichlmeier2014[4]	7.5	Training	1.40
Yamazaki2016[5]	7.5	Training	1.85
Jones2019[6]	7.5	Test	1.40
Zuo2023[7]	2.5	Test	1.10
Chilengi2011[8]	5	Test	1.42
Carmichael2002[9]	15	Test	1.57
		MRD(range)	1.50(1.10-1.85)
		MRD <2	9/9 subjects

Table S5. GMFE values and 95% confidence intervals for predicted and observed AUC_{last} of methotrexate

Reference	Dose [mg]	Dataset	AUC_{pred} [ng·h/l]	AUC_{obs} [ng·h/l]	AUC_{pred}/AUC_{obs}
Kozloski1992[1]	7.5	Training	669.94	601	1.11
Lee2017[2]	7.5	Training	660.13	625	1.06
Namour2012[3]	7.5	Training	665.24	744	0.89
Pichlmeier2014[4]	7.5	Training	572.27	580	0.99
Yamazaki2016[5]	7.5	Training	659.96	620	1.06
Jones2019[6]	7.5	Test	671.03	736	0.91
Zuo2023[7]	2.5	Test	282.29	295	0.96
Chilengi2011[8]	5	Test	530.59	435	1.22
Carmichael2002[9]	15	Test	1339.48	1775	0.75
				GMFE(range)	0.99(0.89-1.10)
				GMFE <2	9/9 subjects

Table S6. GMFE values and 95% confidence intervals for predicted and observed C_{max} of methotrexate

Reference	Dose [mg]	Dataset	Pred C_{max} [ng /l]	Obs C_{max} [ng /l]	(Pred C_{max})/(Obs C_{max})
Kozloski1992[1]	7.5	Training	184.41	173	1.07
Lee2017[2]	7.5	Training	188.57	178	1.06
Namour2012[3]	7.5	Training	126.29	137	0.92
Pichlmeier2014[4]	7.5	Training	187.24	186	1.01
Yamazaki2016[5]	7.5	Training	183.82	156	1.18
Jones2019[6]	7.5	Test	189.99	191	0.99
Zuo2023[7]	2.5	Test	76.31	92.9	0.82
Chilengi2011[8]	5	Test	91.39	72.6	1.26
Carmichael2002[9]	15	Test	384.48	351	1.10
				GMFE(range)	1.04(0.94-1.14)
				GMFE <2	9/9 subjects

Restricted cubic spline model of the maximum blood concentration of methotrexate and dose

Simulated data acquisition

Restricted cubic spline (RCS) models typically necessitate an adequate sample size for robust model construction[17]. In the present study, the clinical data available on methotrexate in healthy subjects (Table S7) were limited. To meet the required modeling standards, the clinical dataset was expanded. The expansion process incorporated the number of subjects (n), the mean maximum plasma concentration (C_{max}), and the standard deviation (SD) for each dose, as reported in the literature. C_{max} values for different doses were further expanded assuming a normal distribution[18]. For studies that provided the coefficient of variation (CV%) and 95% confidence intervals (CIs) for C_{max}, these were converted into SD values based on the sample size. In instances where SD values were not reported for C_{max}, these values were treated as observed data and directly included in the analysis. Additionally, when multiple C_{max} values and corresponding SDs were reported within the same source, data were grouped by literature source and dose. The mean C_{max} and SD were then recalculated for each group. Ultimately, 50 clinical samples were simulated for each dose group in the healthy population to facilitate model construction. Data processing was performed using R version 4.4.1 (R Institute for Statistical Computing, Vienna, Austria)

Table S7. C_{max} data for methotrexate in healthy individuals

**Dose[mg]**	**C_{max}[mg/l]**	**SD**	**References**

7.5	0.173	0.040	Kozloski1992[1]
7.5	0.178	0.040	Lee2017[2]
7.5	0.137	0.003	Namour2012[3]
7.5	0.122	0.002	Namour2012[3]
7.5	0.186	0.004	Pichlmeier2014[4]
15.0	0.303	0.009	Pichlmeier2014[4]
22.5	0.392	0.008	Pichlmeier2014[4]
30.0	0.450	0.018	Pichlmeier2014[4]
7.5	0.156	0.032	Yamazaki2016[5]
7.5	0.191	0.028	Jones2019[6]
2.5	0.093	0.018	Zuo2023[7]
2.5	0.092	0.014	Zuo2023[7]
5.0	0.141	-	Chilengi2011[8]
5.0	0.109	-	Chilengi2011[8]
5.0	0.114	-	Chilengi2011[8]
5.0	0.105	-	Chilengi2011[8]
5.0	0.145	-	Chilengi2011[8]
15.0	0.351	0.120	Carmichael2002[9]

C_{max}: maximum observed plasma concentration; SD: standard deviation

Establishment of the RCS model

The construction of the RCS model was based on the logistic regression framework outlined by Discacciati et al[19]. In this study, both C_{max} and dose were treated as continuous variables, and thus, the ordinary least squares (OLS) method was selected as the most suitable approach to model RCS as a linear regression. Regarding the selection of the model knots, the simulated dataset consisted of variables from six distinct groups. Given that the number of knots typically starts at three or four, as recommended in the literature[19], four knots were chosen for the model, with the knot locations set to their default values. All analyses were conducted using the R package "rms" in R version 4.4.1 (R Institute for Statistical Computing, Vienna, Austria).

RCS model results

The RCS model was developed using 255 clinical simulation samples. Parameter

estimates, standard errors, t-values, and P-values for the model fit are provided in Table S2. All coefficients were found to be statistically significant ($P < 0.0001$). The linear term, sp1(m), demonstrates a positive correlation with the response curve, while the nonlinear terms, sp2(m) and sp3(m), correspond to the acceleration and deceleration effects, respectively.

Table S8. Output results evaluated using a linear regression model with four knots and a restricted cubic spline curve.

Coefficients	Estimate	Standard error	t-value	P-value
(Intercept)	0.0636	0.0074	8.56	<0.0001
sp1(m)[a]	0.0139	0.0014	9.99	<0.0001
sp2(m)	0.0542	0.0121	4.50	<0.0001
sp3(m)	-0.0912	0.0179	-5.10	<0.0001[b]

a When using restricted cubic splines, the first transformation sp1(m) coincides with the Dose itself.

b When restricted cubic splines with four knots are used to model the covariate Dose, a P-value for non-linearity is obtained by testing against the null hypothesis that the parameters of the second and third RCS transformations are equal to 0 (P-value: <0.0001).

Nonlinearity and overall correlation tests ($P < 0.0001$) confirmed the presence of a significant nonlinear dose-response relationship. Figure S3 illustrates the curve

predicting C_{max} as a function of dose, with dose being the independent variable. The plot shows that C_{max} increases sharply at lower doses, but the rate of increase slows at higher doses. This pattern is consistent with the model's predicted results. In particular, sp1(m) and sp2(m) contribute to the curve's acceleration, while sp3(m) predominantly influences its deceleration at higher doses. This outcome is indicative of the pharmacokinetic saturation of methotrexate, aligning with known physiological processes.

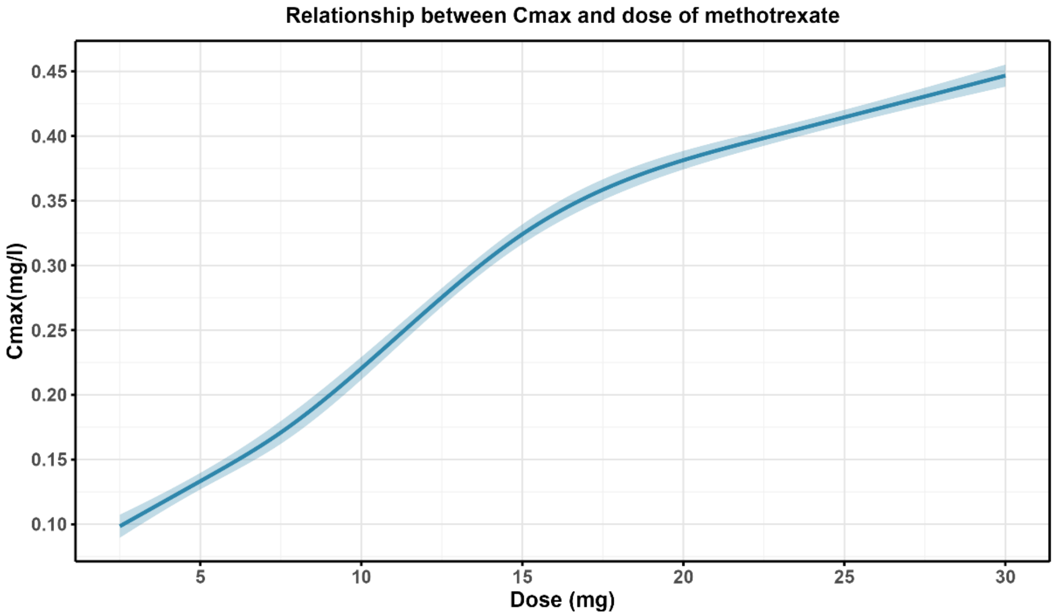

Figure S3. Using the RCS model to investigate the nonlinear relationship between C_{max} and dose of methotrexate, the blue solid line represents the predicted nonlinear relationship between C_{max} and dose; the blue shaded area represents the corresponding confidence interval.

RCS model sensitivity analysis

To evaluate the robustness of the RCS model with respect to knot selection, a

sensitivity analysis was performed by fitting models with varying numbers of knots and comparing their Akaike information criterion (AIC) values. The AIC balances model fit and complexity, with lower values indicating better fit. Figure 2 presents the predicted C_{max} curves for models with different numbers of knots. The 3-knot model (AIC: -993) displays a slightly smoother curve compared to the 4-knot model (AIC: -1017.8), suggesting that it has limited sensitivity due to the fewer knots used to capture the nonlinear relationship between dose and C_{max}. In contrast, the 4-knot model, with additional segments, provides greater flexibility and results in a better fit, as indicated by the lower AIC. Therefore, the 4-knot model was selected as the primary model. The final model results meet the expected validation criteria. The consistent nonlinear trends observed across different models reflect the pharmacokinetic saturation of methotrexate, aligning with pharmacological expectations and enhancing the reliability of the model's predictions.

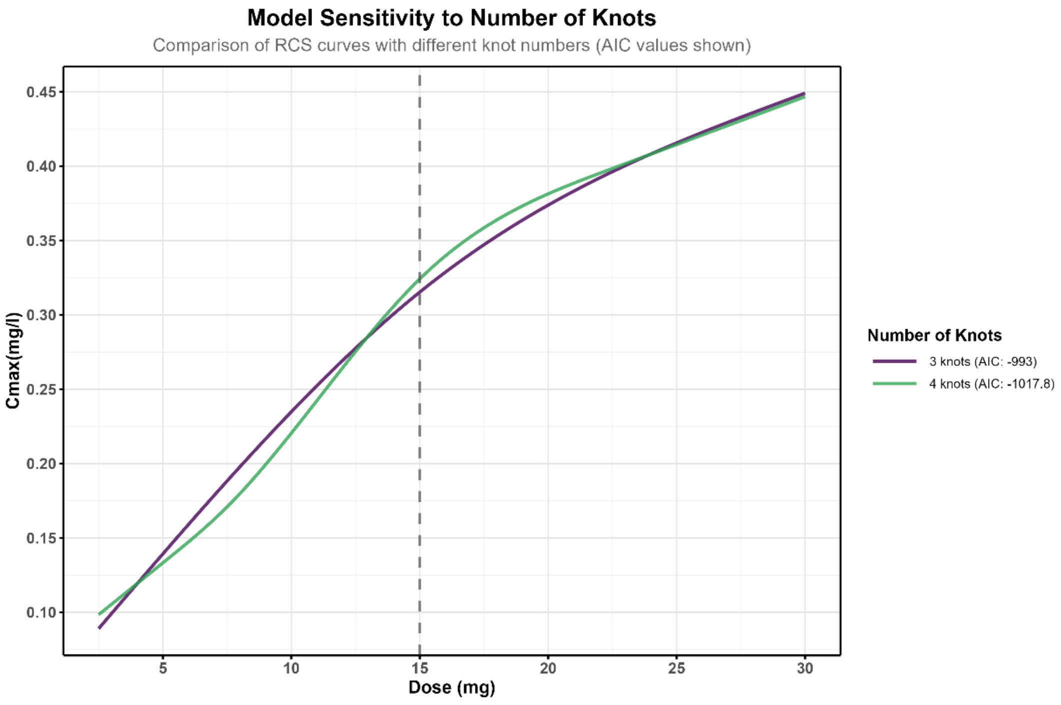

Figure S4. Sensitivity analysis of RCS models with different numbers of knots; the purple curve represents the 3-knot model; the green curve represents the 4-knot model; the vertical dotted line is the median dose reference line.

References

[1] Kozloski, G. D.; De Vito, J. M.; Kisicki, J. C.; Johnson, J. B. The Effect of Food on the Absorption of Methotrexate Sodium Tablets in Healthy Volunteers. *Arthritis & Rheumatism*, **1992**, *35* (7), 761–764. https://doi.org/10.1002/art.1780350708.

[2] Lee, S. K.; Xing, J.; Catlett, I. M.; Adamczyk, R.; Griffies, A.; Liu, A.; Murthy, B.; Nowak, M. Safety, Pharmacokinetics, and Pharmacodynamics of BMS-986142, a Novel Reversible BTK Inhibitor, in Healthy Participants. *Eur. J. Clin. Pharmacol.*, **2017**, *73* (6), 689. https://doi.org/10.1007/s00228-017-2226-2.

[3] Namour, F.; Vanhoutte, F. P.; Beetens, J.; Blockhuys, S.; De Weer, M.; Wigerinck, P. Pharmacokinetics, Safety, and Tolerability of GLPG0259, a Mitogen-Activated Protein Kinase-Activated Protein Kinase 5 (MAPKAPK5) Inhibitor, given as Single and Multiple Doses to Healthy Male Subjects. *Drugs R D*, **2012**, *12* (3), 141–163. https://doi.org/10.2165/11633120-000000000-00000.

[4] Pichlmeier, U.; Heuer, K.-U. Subcutaneous Administration of Methotrexate with a Prefilled Autoinjector Pen Results in a Higher Relative Bioavailability Compared with Oral Administration of Methotrexate. *Clin. Exp. Rheumatol.*, **2014**, *32* (4), 563–571.

[5] Yamazaki, T.; Desai, A.; Goldwater, R.; Han, D.; Lasseter, K. C.; Howieson, C.; Akhtar, S.; Kowalski, D.; Lademacher, C.; Rammelsberg, D.; et al. Pharmacokinetic Interactions Between Isavuconazole and the Drug Transporter Substrates Atorvastatin, Digoxin, Metformin, and Methotrexate in Healthy Subjects. *Clin. Pharmacol. Drug Dev.*, **2017**, *6* (1), 66–75. https://doi.org/10.1002/cpdd.280.

[6] Jones, N. S.; Winter, H.; Katsumoto, T. R.; Florero, M.; Murray, E.; Walker, H.; Singh, N.; Chinn, L. W. Absence of Pharmacokinetic Interactions between the Bruton's Tyrosine Kinase Inhibitor Fenebrutinib and Methotrexate. *J. Pharmacol. Exp. Ther.*, **2019**, *371* (1), 202–207. https://doi.org/10.1124/jpet.119.257089.

[7] Zuo, X.; Zhao, X.; Zhang, T. Pharmacokinetics and Bioequivalence Evaluation of 2 Oral Formulations of Methotrexate Tablets in Healthy Chinese Volunteers under Fasting and Fed Conditions. *Naunyn-schmiedeberg's Arch. Pharmacol.*, **2023**, *396* (4), 803–809. https://doi.org/10.1007/s00210-022-02337-w.

[8] Chilengi, R.; Juma, R.; Abdallah, A. M.; Bashraheil, M.; Lodenyo, H.; Nyakundi, P.; Anabwani, E.; Salim, A.; Mwambingu, G.; Wenwa, E.; et al. A Phase I Trial to Evaluate the Safety and Pharmacokinetics of Low-Dose Methotrexate as an Anti-Malarial Drug in Kenyan Adult Healthy Volunteers. *Malaria Journal*, **2011**, *10* (1), 63. https://doi.org/10.1186/1475-2875-10-63.

[9] Carmichael, S. J.; Beal, J.; Day, R. O.; Tett, S. E. Combination Therapy with Methotrexate and Hydroxychloroquine for Rheumatoid Arthritis Increases Exposure to Methotrexate. *J. Rheumatol.*

[10] Alva-Ensastegui, J. C.; Morales-Avila, E.; de la Luz, A. P.; Bernad-Bernad, M. J. Determination of pKa Values and Deprotonation Order of Methotrexate Using a Combined Experimental-Theoretical Study and Binding Constants of the Methotrexate-Laponite Complex at Different pH Values. *Journal of Photochemistry and Photobiology A: Chemistry*, **2024**, *449*, 115406.

https://doi.org/10.1016/j.jphotochem.2023.115406.
[11] Inoue, K.; Yuasa, H. Molecular Basis for Pharmacokinetics and Pharmacodynamics of Methotrexate in Rheumatoid Arthritis Therapy. *Drug Metab. Pharmacokinet.*, **2014**, *29* (1), 12–19. https://doi.org/10.2133/dmpk.DMPK-13-RV-119.
[12] Takeda, M.; Khamdang, S.; Narikawa, S.; Kimura, H.; Hosoyamada, M.; Cha, S. H.; Sekine, T.; Endou, H. Characterization of Methotrexate Transport and Its Drug Interactions with Human Organic Anion Transporters. *J Pharmacol Exp Ther*, **2002**, *302* (2), 666–671. https://doi.org/10.1124/jpet.102.034330.
[13] Uwai, Y.; Taniguchi, R.; Motohashi, H.; Saito, H.; Okuda, M.; Inui, K. Methotrexate-Loxoprofen Interaction: Involvement of Human Organic Anion Transporters hOAT1 and hOAT3. *Drug Metabolism and Pharmacokinetics*, **2004**, *19* (5), 369–374. https://doi.org/10.2133/dmpk.19.369.
[14] Volk, E. L.; Schneider, E. Wild-Type Breast Cancer Resistance Protein (BCRP/ABCG2) Is a Methotrexate Polyglutamate Transporter. *Cancer Res*, **2003**, *63* (17), 5538–5543.
[15] Chen, Z.-S.; Lee, K.; Walther, S.; Raftogianis, R. B.; Kuwano, M.; Zeng, H.; Kruh, G. D. Analysis of Methotrexate and Folate Transport by Multidrug Resistance Protein 4 (ABCC4).
[16] Michiba, K.; Maeda, K.; Kurimori, K.; Enomoto, T.; Shimomura, O.; Takeuchi, T.; Nishiyama, H.; Oda, T.; Kusuhara, H. Characterization of the Human Intestinal Drug Transport with Ussing Chamber System Incorporating Freshly Isolated Human Jejunum. *Drug Metab Dispos*, **2021**, *49* (1), 84–93. https://doi.org/10.1124/dmd.120.000138.
[17] van der Ploeg, T.; Austin, P. C.; Steyerberg, E. W. Modern Modelling Techniques Are Data Hungry: A Simulation Study for Predicting Dichotomous Endpoints. *BMC Med Res Methodol*, **2014**, *14*, 137. https://doi.org/10.1186/1471-2288-14-137.
[18] Pezoulas, V. C.; Zaridis, D. I.; Mylona, E.; Androutsos, C.; Apostolidis, K.; Tachos, N. S.; Fotiadis, D. I. Synthetic Data Generation Methods in Healthcare: A Review on Open-Source Tools and Methods. *Comput Struct Biotechnol J*, **2024**, *23*, 2892–2910. https://doi.org/10.1016/j.csbj.2024.07.005.
[19] Discacciati, A.; Palazzolo, M. G.; Park, J.-G.; Melloni, G. E. M.; Murphy, S. A.; Bellavia, A. Estimating and Presenting Non-Linear Associations with Restricted Cubic Splines. *International Journal of Epidemiology*, **2025**, *54* (4), dyaf088. https://doi.org/10.1093/ije/dyaf088.
